# Correlation among photoluminescence and the electronic and atomic structures of Sr_2_SiO_4_:xEu^3+^ phosphors: X-ray absorption and emission studies

**DOI:** 10.1038/s41598-020-69428-7

**Published:** 2020-07-29

**Authors:** Shi-Yan Zheng, Jau-Wern Chiou, Yueh-Han Li, Cheng-Fu Yang, Sekhar Chandra Ray, Kuan-Hung Chen, Chun-Yu Chang, Abhijeet R. Shelke, Hsiao-Tsu Wang, Ping-Hung Yeh, Chun-Yen Lai, Shang-Hsien Hsieh, Chih-Wen Pao, Jeng-Lung Chen, Jyh-Fu Lee, Huang-Ming Tsai, Huang-Wen Fu, Chih-Yu Hua, Hong-Ji Lin, Chien-Te Chen, Way-Faung Pong

**Affiliations:** 1 Department of Physics, Tamkang University, Tamsui, 251 Taiwan, ROC; 2 College of Physics and Information Engineering, Quanzhou Normal University, Quanzhou, 362000 Fujian People’s Republic of China; 3 Department of Applied Physics, National University of Kaohsiung, Kaohsiung, 811 Taiwan, ROC; 4 Department of Chemical and Material Engineering, National University of Kaohsiung, Kaohsiung, 811 Taiwan, ROC; 5 Department of Physics,CSET, University of South Africa, Johannesburg, 1710 South Africa; 6 Department of Material Science and Engineering, National Chiao Tung University, Hsinchu, 300 Taiwan, ROC; 7 National Synchrotron Radiation Research Center, Hsinchu, 300 Taiwan, ROC

**Keywords:** Materials science, Physics

## Abstract

A series of Eu^3+^-activated strontium silicate phosphors, Sr_2_SiO_4_:xEu^3+^ (SSO:xEu^3+^, x = 1.0, 2.0 and 5.0%), were synthesized by a sol–gel method, and their crystalline structures, photoluminescence (PL) behaviors, electronic/atomic structures and bandgap properties were studied. The correlation among these characteristics was further established. X-ray powder diffraction analysis revealed the formation of mixed orthorhombic α'-SSO and monoclinic β-SSO phases of the SSO:xEu^3+^ phosphors. When SSO:xEu^3+^ phosphors are excited under ultraviolet (UV) light (λ = 250 nm, ~ 4.96 eV), they emit yellow (~ 590 nm), orange (~ 613 nm) and red (~ 652 and 703 nm) PL bands. These PL emissions typically correspond to 4*f*–4*f* electronic transitions that involve the multiple excited ^5^D_0_ → ^7^F_J_ levels (J = 1, 2, 3 and 4) of Eu^3+^ activators in the host matrix. This mechanism of PL in the SSO:xEu^3+^ phosphors is strongly related to the local electronic/atomic structures of the Eu^3+^–O^2−^ associations and the bandgap of the host lattice, as verified by Sr *K*-edge and Eu *L*_3_-edge X-ray absorption near-edge structure (XANES)/extended X-ray absorption fine structure, O *K*-edge XANES and *K*_α_ X-ray emission spectroscopy. In the synthesis of SSO:xEu^3+^ phosphors, interstitial Eu_2_O_3_-like structures are observed in the host matrix that act as donors, providing electrons that are nonradiatively transferred from the Eu 5*d* and/or O 2*p*–Eu 4*f/*5*d* states (mostly the O 2*p*–Eu 5*d* states) to the ^5^D_0_ levels, facilitating the recombination of electrons that have transitioned from the ^5^D_0_ level to the ^7^F_J_ level in the bandgap. This mechanism is primarily responsible for the enhancement of PL emissions in the SSO:xEu^3+^ phosphors. This PL-related behavior indicates that SSO:xEu^3+^ phosphors are good light-conversion phosphor candidates for use in near-UV chips and can be very effective in UV-based light-emitting diodes.

## Introduction

Rare-earth ion-doped inorganic phosphors are luminescent materials with practical applications in many devices, including white light-emitting diodes (LEDs), multicolor sensors, high-density optical storage devices and devices for detecting high-energy radiation^1, 2^. Among the rare-earth elements, europium (Eu) is of special interest as a dopant because it exhibits valence fluctuation between divalent (Eu^2+^) and trivalent (Eu^3+^) states, remarkably efficient phosphors and narrow-band emission properties that cause it to function as an emission center in a host lattice^3^. The coordination environment and the type of crystals determine the valence state of activators and affect the photoluminescence (PL) properties of phosphors in which they are incorporated^3^. These materials, when excited by light of a suitable wavelength/energy, provide a high PL yield and favorable chromaticity owing to the intra-4*f*–4*f* parity-forbidden transitions of the Eu^3+^ activators or the 4*f–*5*d* transitions of the Eu^2+^ rare-earth ions, whose intensity depends on the site symmetry and the nature of the host matrix^4^. Eu^3+^ dopants typically exhibit orange-red (^5^D_0_ → ^7^F_J_), green (^5^D_1_ → ^7^F_1_) or blue (^5^D_2_ → ^7^F_0_) luminescence as a result of intra 4*f*–4*f* transitions^5,6^. Furthermore, the emission of the Eu^2+^ dopant originates from the 4*f–*5*d* transition, the color of which varies from ultraviolet (UV) to red, depending on the crystal symmetry and the effect of the crystal field on the excited 5*d* states^7,8^. Notably, in Eu^3+^ emission, the resonant excitation cross-section of 4*f*–4*f* transitions is normally small^1, 6^, so emission occurs upon transition to the ground ^7^F_J_ state from ^5^D_J_ states, presumably involving the O^2−^ → Eu^3+^ charge-transfer (CT) band^7,8^. The replacement of divalent ions in the host lattice with trivalent ions requires charge compensation to maintain electrical neutrality and plays a critical role in the excitation of Eu^3+^-activators in which nonradiative relaxation to ^5^D_J_ states is followed by radiative emission^9,10^.

Among various host oxides for phosphors, strontium silicate, Sr_2_SiO_4_ (SSO), is an excellent host material owing to its stable crystal structure, high mechanical strength and thermal stability, which is provided by the tetrahedral silicate (SiO_4_)^4−^ host matrix,^11^ and it has potential application in the development of white LEDs. The sites of Sr^2+^ ions are of two types: (i) highly symmetric ten-coordinate sites (Sr_1_ sites) and (ii) less symmetric nine-coordinate sites (Sr_2_ sites). When this silicate host is doped with Eu^3+^ ions, the trivalent-metal ions may be distributed/substituted among these two types of Sr sites. The luminescence of Eu^3+^ activators depends strongly on the site symmetries^12^. Depending on whether they are at high-symmetry (Sr_1_ sites) or low-symmetry (Sr_2_ sites) sites in the host matrix, Eu^3+^ activators generally emit red or orange light^3^. Doping with trivalent Eu^3+^ ions changes the local charge distribution and generates interstitial defects or vancancies^13–15^ that act as donor levels and thus increase the PL. Previous studies^16–19^ have suggested that the substitution of cations by Eu in the host matrix is an effective means of adjusting the PL of phosphor materials. Intuitively, Eu^3+^ ions that replace Sr^2+^ ions in the SSO lattice can be readily dissolved because the ionic radii of nine-coordinate Eu^3+^ (~ 1.30 Å) and ten-coordinate Eu^3+^ (~ 1.35 Å) are very close to those of nine-coordinate Sr^2+^ (~ 1.31 Å) and ten-coordinate Sr^2+^ (~ 1.36 Å)^1,3,20^, respectively. Additionally, SSO is a unique lattice in which the O^2−^ → Eu^3+^ CT band lies much lower than that in other host lattices^9^. Therefore, in SSO:Eu^3+^ phosphors, Eu^3+^ ions are incorporated into the host lattice probably as substitutes for Sr^2+^ ions, so the charge becomes imbalanced; upon neutralization, the O^2−^ charge-compensating agent occupies the sites of defects, so the local environment of Eu^3+^ in the crystal field becomes more symmetrical, thus increasing the intensity of the PL^3^. Yellow emission (^5^D_0_ → ^7^F_1_) dominates when the site of the Eu^3+^ ions exhibits high symmetry, while orange-red emission (between ^5^D_0_ → ^7^F_2_ and ^5^D_0_ → ^7^F_4_) dominates when the site does not exhibit symmetry. An earlier study^21^ reported unusual luminescence spectra of Eu-doped SSO, with a strong ^5^D_0_ → ^7^F_0_ line at ~ 575 nm, although whether the 575 nm emission arises from the ^5^D_0_ → ^7^F_0_ transition is ambiguous^1^. This transition normally occurs when the site symmetry is very low. The abnormally high energy of emission in that study was postulated to result from the presence of extra interstitial O^2−^ ligands that were not bound to silicon but formed an association (Eu^3+^–O^2−^)^1,3,5,18^. An O^2−^ → Eu^3+^ CT band at such long wavelengths in oxides is peculiar and is still debated. Eu typically yields very intense and wide visible emissions, which can be attributed to electric-dipole-allowed transitions from the Eu 5*d* to 4*f* energy in phosphors and related materials^15,22–24^. Changes in the local environments and control the Eu valence states (Eu^3+^ and Eu^2+^) in phosphor materials to tune their activation have been extensively investigated with the aim of improving the materials’ PL properties^3,25^. The PL mechanism of the Eu-doped SSO matrix is still debated despite the numerous studies mentioned above. However, the local environments of the Eu activators in the SSO host matrix have not been comprehensively investigated, and in particular, no direct experimental observation of the correlation among PL behavior, the electronic/atomic structures of Eu ions, and the bandgap of the host matrix has been reported. In this study, an alkaline earth silicate host doped with Eu ions (SSO:xEu^3+^ phosphors, x = 1.0, 2.0 and 5.0%) in the α′-phase and the β-phase at various Eu^3+^ concentrations is synthesized using the sol–gel process^26^. The effect of Eu^3+^ activators on the PL properties, particularly the correlation among the PL emission, the local electronic/atomic structures and the energy bandgap of the SSO:xEu^3+^ phosphors, is studied using synchrotron-based X-ray absorption near-edge structure (XANES), extended X-ray absorption fine structure (EXAFS) and X-ray emission spectroscopy (XES) techniques. The aim of this work is to elucidate the PL emissions that originate from interstitial Eu_2_O_3_-like structures in the host matrix. These structures are primarily responsible for the nonradiative electron transfer from Eu 5*d* states and/or O 2*p*–Eu 4*f/*5*d* states (mostly O 2*p*–Eu 5*d* states) to ^5^D_0_ levels, facilitating the 4*f*–4*f* electronic transitions from the excited ^5^D_0_ → ^7^F_J_ levels in the band gap of the host matrix. Therefore, the overall PL intensity increases with Eu concentration in the SSO:xEu^3+^ phosphors.

## Results and discussion

**Figure 1:**
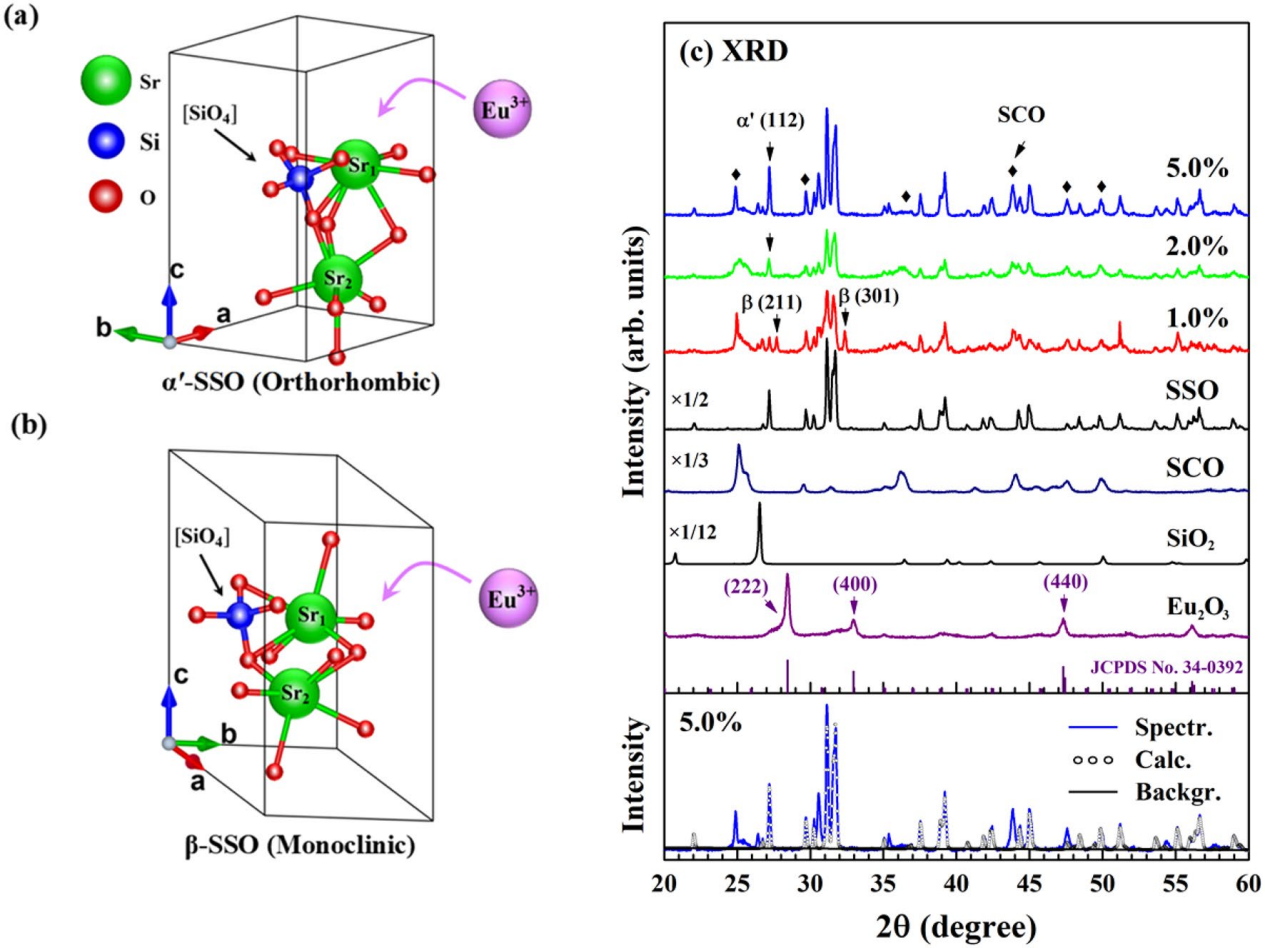
Atomic structure of (**a**) the α′-SSO phase (orthorhombic) and (**b**) the β-SSO phase (monoclinic) upon Eu^3+^-doping in the SSO host matrix [Drawn using VESTA software after considering the SSO raw crystallographic files (https://www.materialsproject.org/)]; (**c**) XRD patterns of synthesized SSO:xEu^3+^ phosphors with various concentrations of Eu^3+^ ions and reference spectra of SSO, SCO, SiO_2_ and Eu_2_O_3_ with the JCPDS pattern. The bottom panel shows XRD data for the SSO:xEu^3+^ phosphor (x = 5.0%) and calculations of the crystal structure made using GSAS software.

Figure 1a,b shows the atomic structures of the (orthorhombic) α′ and (monoclinic) β phases of the SSO matrix, respectively. As the Eu^3+^ doping concentration increases, β-phase SSO is likely converted into α′-phase SSO. The structure of SSO is similar to the non-close-packed structure of K_2_SiO_4_^27^. As displayed in Fig. 1a,b, one unit cell of SSO comprises 26 atoms that share four formula units. Its structure can be best described as comprising corner-sharing SiO_4_ tetrahedra in parallel chains. The oxygen ions are located at three types of nonequivalent lattice sites, and the Si ions are located at the center of the oxygen tetrahedron. When Eu^3+^ is doped into the SSO matrix, the Eu^3+^ ions may affect the SSO host lattice by changing the lattice constants and/or causing structural distortion, varying the ratio of the α′ phase to the β phase in the SSO matrix^18^. However, this modification of the SSO matrix and/or site occupancy can only be observed by X-ray diffraction (XRD) at a rather high Eu^3+^-dopant concentration (≥ 5%) in the host matrix^18^. Figure 1c presents XRD patterns of both the as-synthesized SSO:xEu^3+^ phosphors with various concentrations of Eu^3+^ ions and SSO, SrCO_3_ (SCO), SiO_2_ and Eu_2_O_3_ for reference. The diffraction peak at (112) typically corresponds to the α*′*-SSO:xEu^3+^ phase, and the (211) and (301) peaks typically correspond to the β-SSO:xEu^3+^ phase. This result clearly shows that the crystal structures of the SSO:xEu^3+^ phosphors and the SSO host were mixed phases of both the α′ and β-SSO:xEu^3+^ phases. Prominent (211) and (301) diffraction peaks were observed at an Eu^3+^ ion concentration of 1.0%, whereas the (112) peak became more prominent as the concentration of Eu^3+^ ion increased above 1.0%, as was particularly evident for x = 5.0% and pure SSO. Apparently, as the Eu^3+^ doping concentration increased, the SSO matrix became increasingly α′-rich SSO:xEu^3+^ phase from β-rich SSO:xEu^3+^^28^. Figure 1c also shows a characteristic (222) diffraction peak at ~ 28.2° that arises from the Eu_2_O_3_ phase. This peak is identified as the cubic phase of Eu_2_O_3_ and confirmed by comparison with the JCPDS pattern (card No. 34-0392). Apparently, XRD results show that doping with Eu^3+^ ions does not significantly change the structure of the lattice. The peaks that are marked with ‘♦’ in the XRD spectra indicate the presence of a tiny amount of SCO in the SSO:xEu^3+^ phosphors. The variation in the weight ratio of α′-SSO/β-SSO phases with the concentration of Eu^3+^ ions in the SSO:xEu^3+^ phosphors and SSO was quantitatively analyzed. The analysis was based on the ratio of α′-SSO to β-SSO phase peaks and was performed using general structure analysis system (GSAS) software^29^. To qualitatively study the effect of Eu^3+^ concentration on the crystal structure and the weight ratio of α′-SSO/β-SSO phases in the SSO:xEu^3+^ phosphors, XRD spectra of the SSO:xEu^3+^ phosphors were simulated by GSAS software, and the simulation of the x = 5.0% sample is presented in the bottom panel of Fig. 1c, along with the experimental results. Table 1 presents the weight percentages and ratios of α′- and β-SSO phases in the SSO:xEu^3+^ phosphor and compares them with those reported elsewhere^19^. Table 1 indicates that the α′-SSO/β-SSO phase ratio was 0.70 at a Eu^3+^ ion concentration of x = 1.0% and increased to 8.65 and 16.93 at Eu^3+^ ion concentrations of x = 2.0 and 5.0%, respectively. However, the SSO host matrix had the highest (26.86) α′-SSO/β-SSO phase ratio. These results further confirm the formation of α′-rich SSO:xEu^3+^ phosphors at higher Eu^3+^-doping concentrations in the SSO:xEu^3+^ phosphors, which are associated with a more symmetric structure in the more highly Eu^3+^-doped samples. It is noted that the α′-SSO/β-SSO phase ratios of 1.0% Eu^3+^-doped SSO and pure SSO are unlikely not close to each other. The results differ because SSO:xEu^3+^ and pure SSO are synthesized separately in situ using the corresponding precursor; *i.e.*, the SSO:xEu^3+^ phosphors were not a straight mixture of SSO and Eu_2_O_3_, although in both cases, the synthesis process and temperatures remain the same. Table 2 shows the lattice constants (***a***, ***b ***and ***c***) of the α′-SSO and β-SSO phases, the crystal angle θ in the β-SSO phase and the volumes of both the α′- and β-SSO phases in the SSO:xEu^3+^ phosphors. The parameters in Table 2 do not vary significantly with Eu^3+^ doping in the SSO: xEu^3+^ phosphors, suggesting that the Eu^3+^ ions may/may not substitute at Sr sites in the SSO host matrix, as discussed above: not only do the nine-/ten-coordinate Eu^3+^ ions have ionic radii comparable to those of the corresponding Sr in the host matrix, but also the atomic radii of Eu (2.56 Å) and Sr (2.45 Å) atoms are similar to each other^30^. In particular, no clear XRD patterns of Eu_2_O_3_-like structures in SSO:xEu^3+^ phosphors were observed, as shown in Fig. 1c, and this result can be explained by the fact that the Eu^3+^ doping concentration in the SSO host matrix is too low to be detected by XRD. However, high-resolution transmission electron microscopy (HRTEM) clearly shows the cubic Eu_2_O_3_-phase in the SSO: xEu^3+^ matrix, as presented in the lower inset of Fig. 4a, and will be discussed later.

**Table 1:** Weight percentages of β-SSO and α′-SSO phases and α′-SSO/β-SSO phase ratio.

Sample	β-SSO	α′-SSO	α′-SSO/β-SSO
1.0%	58.89 ± 0.03	40.90 ± 0.02	0.70
2.0%	10.06 ± 0.03	87.02 ± 0.02	8.65
5.0%	5.39 ± 0.03	91.27 ± 0.02	16.93
SSO	3.59 ± 0.03	96.41 ± 0.02	26.86

**Table 2:** Lattice constants (***a***, ***b ***and ***c***) obtained by fitting with the β-SSO and α′-SSO phases, crystal angle θ in the β-SSO phase and volumes for both the β- and α′-SSO:xEu^3+^ phases.

Sample	Fitting with β-SSO					Fitting with α′-SSO			
	*a* (Å)	*b* (Å)	*c* (Å)	θ (degree)	Volume (Å^3^)	*a* (Å)	*b* (Å)	*c* (Å)	Volume (Å^3^)
1.0%	5.661 ± 0.002	7.084 ± 0.002	9.745 ± 0.002	92.61 ± 0.03	390.4 ± 0.2	5.663 ± 0.001	7.071 ± 0.001	9.737 ± 0.001	390.0 ± 0.2
2.0%	5.657 ± 0.002	7.065 ± 0.002	9.748 ± 0.002	92.57 ± 0.03	389.2 ± 0.2	5.662 ± 0.001	7.074 ± 0.001	9.736 ± 0.001	390.0 ± 0.2
5.0%	5.683 ± 0.002	7.095 ± 0.002	9.726 ± 0.002	92.45 ± 0.03	391.8 ± 0.2	5.653 ± 0.001	7.077 ± 0.001	9.727 ± 0.001	389.2 ± 0.2
SSO	5.658 ± 0.002	7.087 ± 0.002	9.779 ± 0.002	92.60 ± 0.03	391.7 ± 0.2	5.668 ± 0.001	7.079 ± 0.001	9.742 ± 0.001	390.9 ± 0.2

**Figure 2:**
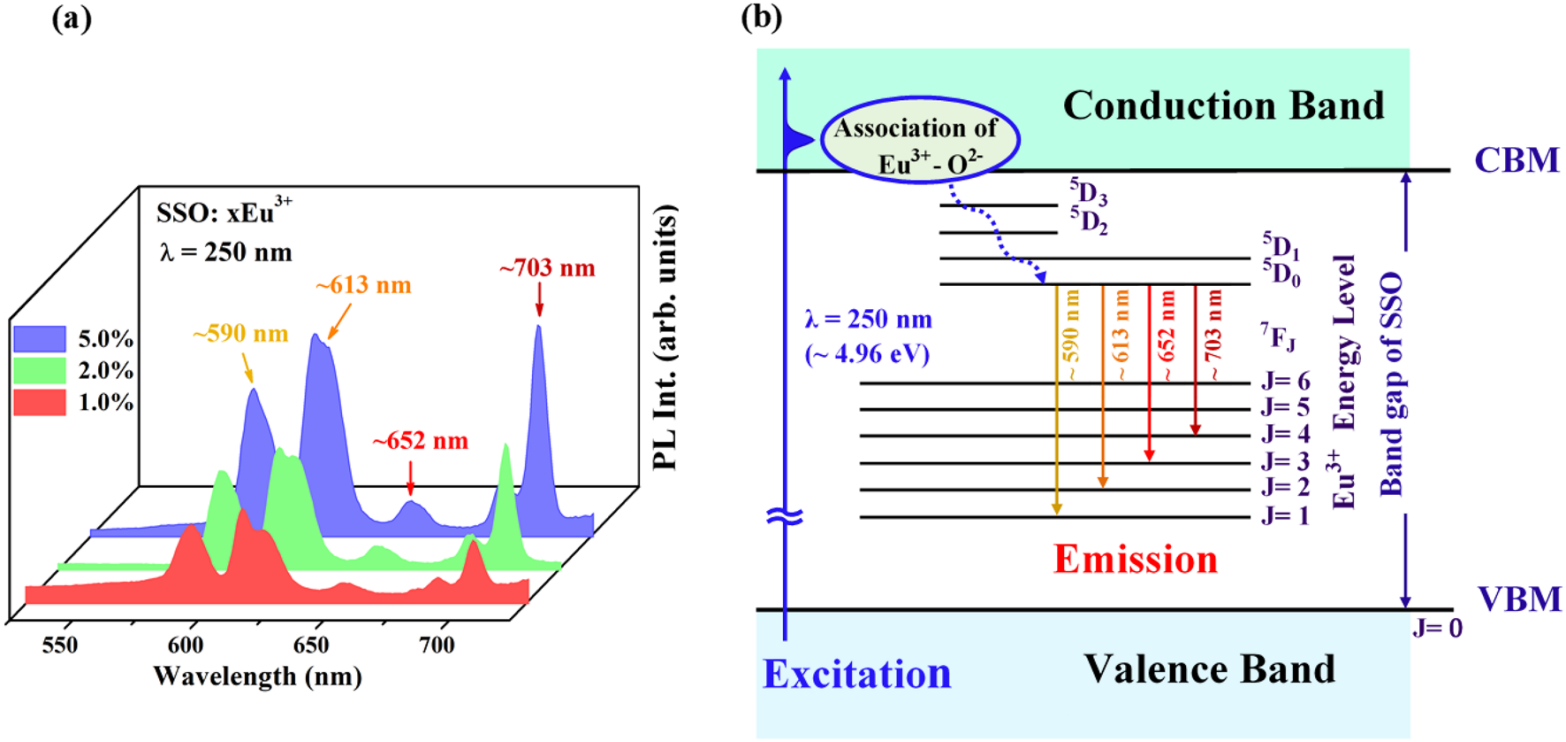
(**a**) PL spectra of SSO:xEu^3+^ phosphors with sharp lines centered at ~ 590, 613, 652 and 703 nm. (**b**) Energy level scheme of the Eu^3+^-activator and a sketch of the excitation of electrons from VB/VBM to CB/CBM upon excitation by UV light (λ = 250 nm, ~ 4.96 eV) with energies that exceed the energy gap in the SSO:xEu^3+^ phosphors. The process also involves transfer of electrons from the Eu^3+^–O^2−^ associations to the ^5^D_0_ level (dashed line), corresponding to PL spectra of the ^5^D_0_ → ^7^F_J_ transitions (J = 1, 2, 3 and 4), as observed in (**a**).

Figure 2a displays the PL spectra of SSO:xEu^3+^ phosphors recorded at room temperature upon excitation at a wavelength (λ) of 250 nm (~ 4.96 eV). The PL spectra include rather broad features that are centered at ~ 590, 613, 652 and 703 nm, consistent with previously reported results^28^. The PL spectral features reveal emissions from Eu^3+^ activators in the host matrix; the emission features are related to the intra-4*f*–4*f *^5^D_0_ → ^7^F_J_ (J = 1, 2, 3 and 4) electronic transitions. The observed ^5^D_0_ → ^7^F_1_ (^7^F_3_) transitions at ~ 590 (652) nm and ^5^D_0_ → ^7^F_2_ (^7^F_4_) transitions at ~ 613 (703) nm are special; the first is a symmetry-sensitive transition and is known as the magnetic dipole transition (MDT) with the selection rule (ΔJ = 0, ± 1), and the second is a hypersensitive electric dipole transition (EDT) with ΔJ ≥ 2^18^. Both are sensitive to the local environment and depend on the symmetry of the crystal field of the Eu^3+^ activators in the host matrix^3,9^. Typically, the most important parameter for understanding symmetry is the asymmetric ratio or asymmetric factor (I_rat_ = I_EDT_/I_MDT_), which is defined as the ratio of the integral intensity of EDT (^5^D_0_ → ^7^F_2,4_) to that of MDT (^5^D_0_ → ^7^F_1,3_). At a crystal site with inversion symmetry, MDT is usually the most intense emission feature, whereas at a site without inversion symmetry, EDT is the strongest emission feature because transitions with ΔJ =  ± 2 are hypersensitive to small deviations from inversion symmetry.^18^ The relative intensity I_EDT_/I_MDT_ strongly depends on the local symmetry around the Eu^3+^ activators and thus provides information about the degree of distortion from inversion symmetry of the local environment around the Eu^3+^ activators in the host matrix. A lower symmetry around Eu^3+^ activators results in a higher asymmetric ratio or asymmetric factor. In this work, I_rat_ [I(^5^D_0_ → ^7^F_2_)/I(^5^D_0_ → ^7^F_1_)], which is the ratio of the integrated intensities in the regions 603 to 634 and 577 to 601 nm, changed from 1.29 (x = 1.0%) to 1.57 (x = 2.0%) to 1.61 (x = 5.0%) as the asymmetric field around the Eu^3+^ activators increased, reflecting a small change in the lattice distortion, possibly caused by either substitution at the Sr sites or the formation of interstitial Eu–O associations in the SSO:xEu^3+^ phosphors. Yin et al.^31^ observed the PL properties of a Eu_2_O_3_/Tb_2_O_3_ film that was deposited on a *p*^+^-type Si substrate with a small lattice mismatch using ITO and Ag as the negative and positive electrodes, respectively. These excitations (^5^D_0_ → ^7^F_1_ at 583 nm and ^5^D_0_ → ^7^F_2_ at 611 nm) yielded relatively broad-band features and improved the I_rat_, which can be associated with the CT band, owing to the injection of electrons and holes into the unoccupied Eu 5*d* and occupied O 2*p* states of the Eu_2_O_3_ layer from ITO and Tb_2_O_3_ films, respectively, thus enhancing the 4*f*–4*f* electronic transitions. The transition from ^5^D_0_ → ^7^F_0_ (J = 0) is forbidden by both ED and MD, and only weak transitions are observed as a result of the crystal field-induced J-mixing effect of the Eu^3+^ activators. However, this transition (^5^D_0_ → ^7^F_0_) occurs because of the unique structural features of the orthorhombic polymorph of SSO.^1,9^ In the atomic structure of SSO, as shown in Fig. 1a,b, the Si atoms in the SiO_4_ tetrahedra form a parallel chain. The Sr_1_ sites form more symmetric linear chains of Si–O–Sr_1_–O–Sr_2_, but the Sr_2_ sites form less symmetric zig-zag chains of Sr_1_–O–Sr_2_–O–Sr_1_ along the ***b***-axis.^1,3^ In the present case, the ^5^D_0_ → ^7^F_0_ transition (~ 575 nm) is completely absent, suggesting that the Eu^3+^ activators have rather asymmetric environments in the SSO:xEu^3+^ phosphors. Notably, the total PL intensity in Fig. 2a is proportional to the ratio of α′-SSO/β-SSO phases, as presented in Table 1. Based on the above discussion, the enhanced PL emission is associated with not only the α′-SSO/β-SSO phase ratio (or the degree of symmetry of Eu^3+^ activators) but also the effect on the CT band of the Eu–O associations in the SSO:xEu^3+^ phosphors described here.

The PL of selected rare-earth ions that are doped into host matrices is well known to be able to be used as a spectral probe of crystal structure, which is closely related to the crystal field of the activators and is determined by the valence-band maximum (VBM) and conduction-band minimum (CBM) of the matrix, as well as the partial electronic density of states (DOSs) of the luminescent centers and intrinsic/extrinsic defects, which function as trap centers in the matrix.^9^ The Eu^3+^ ion has six electrons in the 4*f* shell, which is not an entirely filled *f* orbital. Figure 2b presents a typical energy diagram of Eu 4*f*–4*f* electronic transitions, and the Eu^3+^ activators usually comprise emission features in the red spectral region, where the emission transition is caused by the crystal field splitting of the ^7^F_J_ levels. In addition to these emission features, emissions from higher ^5^D levels, viz. ^5^D_1_ (green), ^5^D_2_ (blue) and even ^5^D_3_, are commonly observed. However, their presence or absence depends principally on the host lattice.^9^ Nevertheless, in this study of the origin of the Eu^3+^ luminescence of SSO:xEu^3+^ phosphors, the emission features mostly correspond to transitions from the excited ^5^D_0_ level to the ^7^F_J_ levels in the Eu 4*f*^6^ configuration and are affected strongly by the CT band and/or the local electronic/atomic structures of the Eu^3+^ ions in the formation of the Eu^3+^–O^2−^ associations and the host lattice. Principally, the PL is a three-step process: (1) absorption of a UV photon, (2) transfer of the excitation energy (or electrons) to the luminescent centers, and (3) radiative emission from the luminescent centers. To illustrate the effects of the luminescent centers of Eu^3+^ activators that are involved in the correlation between the CT band and the Eu^3+^–O^2−^ associations and of the bandgap of the host lattice on the absorption/excitation and emission processes, Fig. 2b schematically depicts the excitation of electrons from the valence band (VB) to the conduction band (CB) of the host matrix upon excitation by UV light (λ = 250 nm, ~ 4.96 eV); the excitation by UV light presumably exceeds the energy gap (the energy separation between the VBM and the CBM), E_g_, of the SSO:xEu^3+^ phosphors because the bandgap of the α′-phase/β-phase SSO is close to 4.49/4.11 eV^19^, and that of the mixed α′- and β-phases of SSO is close to 4.12 eV^32^, yielding free electrons and holes in the CB and VB, respectively. This process also involves the relative energy levels of Eu^3+^–O^2−^ associations, since the valence electrons of the activators/Eu^3+^–O^2−^ associations can also be excited directly by UV or by energy that is transferred from the host lattice, so the overall excited free electrons above/near the CBM thermally cross and relax non-radiatively (as indicated by the dashed line), being transferred to the lower excited state of ^5^D_0_. This process is followed by de-excitation to the ground states via radiative electronic transitions, corresponding to the PL from the ^5^D_0_ → ^7^F_J_ transitions (J = 1, 2, 3 and 4), which are yellow (~ 590 nm), orange (~ 613 nm) and red (~ 652 nm and 703 nm), respectively, as observed in Fig. 2a. Understandably, the position of the PL spectral features does not change significantly with the Eu^3+^ doping concentration, but their overall intensity increases. The enhancement of PL is related to the transfer of electrons from the CT band, which is related to the Eu^3+^–O^2−^ associations. Hypothetically, the Eu^3+^–O^2−^ associations provide extra electrons that are transferred from Eu^3+^–O^2−^ states with free electrons as a result of excitation from VB, resulting in nonradiative transfer to lower ^5^D_0_ levels.

**Figure 3:**
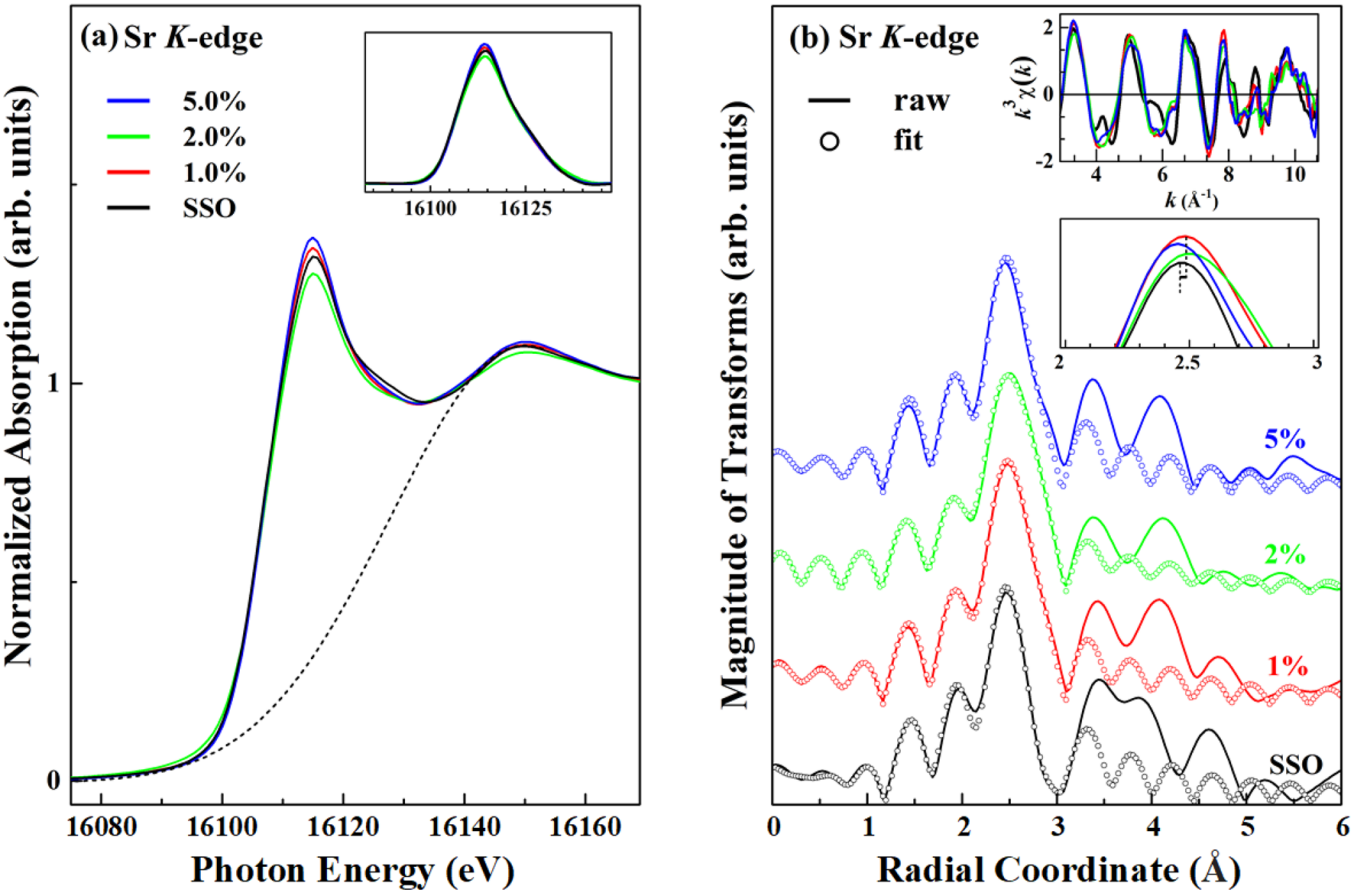
(**a**) Normalized Sr *K*-edge XANES spectra of SSO:xEu^3+^ phosphors (x = 1.0, 2.0 and 5.0%) and reference SSO. The inset shows a magnification of the near-edge feature from 16,080 to 16,140 eV after subtracting the Gaussian background from the near-edge feature. (**b**) FT of EXAFS in *R*-space of SSO:xEu^3+^ phosphors and SSO. The solid profile was obtained from raw data, whereas the circular marks represent the best fit for the first coordination shell. The upper and lower insets present Sr *K*-edge EXAFS *k*^3^χ data and magnifications of the first main FT spectra of SSO:xEu^3+^ phosphors and SSO, respectively.

Figure 3a displays the normalized Sr *K*-edge XANES spectra of SSO:xEu^3+^ phosphors (x = 1.0, 2.0 and 5.0%) and the reference SSO obtained in bulk-sensitive transmission mode. The inset shows magnification of the near-edge features from ~ 16,080 to 16,140 eV after subtraction of the Gaussian background from the main near-edge feature, as represented by the dashed line in Fig. 3a. By the dipole-transition selection law, the main absorption near-edge feature of SSO:xEu^3+^ phosphors at the Sr *K*-edge represents the transition of electrons from Sr 1* s* to 5*p* unoccupied states and is slightly more (x = 1.0 and 5.0%) or less (x = 2.0%) intense than that of the reference SSO, suggesting no significant change in the electronic unoccupied Sr 5*p* states of Eu-doped SSO:xEu^3+^ phosphors relative to that of SSO. Figure 3b shows the magnitudes of the Fourier transformed (FT) Sr *K*-edge EXAFS of the SSO:xEu^3+^ phosphors and SSO. The upper inset shows the corresponding *k*^3^-weighted *k*^3^χ oscillating spectra. The selected *k*-range for the FT spectra was ~ 2.9–10.7 Å^−1^. To provide more comprehensive insight into the local atomic structures at the Sr_1_ and Sr_2_ sites as the Eu concentration in the SSO:xEu^3+^ phosphors increases, the nearest-neighbor (NN) coordination number (N_1_/N_2_), Sr_1_–O/Sr_2_–O bond length (R_1_/R_2_), and mean square fluctuation of the Debye–Waller factor (DWF, σ_1_^2^/σ_2_^2^) at the Sr_1_/Sr_2_ sites were obtained by fitting Sr *K*-edge EXAFS spectra. All spectra were analyzed by standard procedures using the Athena program package^33,34^ to extract quantitative local information about the local atomic structures at the Sr_1_/Sr_2_ sites in the SSO:xEu^3+^ phosphors and SSO. Table 3 presents the fitting results for the first shell (mixing NN Sr_1_–O and Sr_2_–O bond lengths) of the SSO:xEu^3+^ phosphors and SSO. The results of the analysis in Table 3 clearly indicate that the local atomic structures (N_1_/N_2_, R_1_/R_2_ and σ_1_^2^/σ_2_^2^) at the Sr_1_/Sr_2_ sites in the matrix with various Eu^3+^ doping concentrations remain nearly unchanged, revealing that the SSO:xEu^3+^ phosphors easily tolerate Eu incorporation without significant distortion of the host lattice. Clearly, the coordination number of N_1_/N_2_, the NN Sr_1_–O/Sr_2_–O bond length and the corresponding DWF σ_1_^2^/σ_2_^2^ in the SSO:xEu^3+^ phosphors and SSO are also very similar, although the former have slightly longer bond lengths and a smaller DWF than the latter [the slight shift in the magnified scale of the over plotting first main FT features is easily observed in the lower inset of Fig. 3b]. With reference to Fig. 3a, b, the similarity of the general line shapes in the XANES/the first main FT spectral feature at the Sr *K*-edge of the SSO:xEu^3+^ phosphors relative to those of the SSO host reveals that Eu^3+^-doping in the SSO:xEu^3+^ phosphors does not significantly distort the local electronic and atomic structures at the Sr sites in the matrix. Since Eu^3+^ does not replace the Sr site and exists in the host lattice, the host lattice provides space to accommodate Eu^3+^ ions in the SSO:xEu^3+^ matrix. Importantly, these results suggest that the Eu^3+^ activators may not substitute at both/either the Sr_1_ and/or the Sr_2_ sites, so the CT band of O^2−^ → Eu^3+^ does not initially undergo electron transfer by polyhedral SrO_10_/SrO_9_ (Sr_1_–O/Sr_2_–O → Eu) in the SSO:xEu^3+^ phosphors^1,3,16^. Furthermore, as shown in the lower inset of Fig. 3b, the intensities of the first main feature (NN Sr–O bond length) in the FT spectra of the SSO:xEu^3+^ phosphors overall exceed that of the SSO, primarily because the former contains fewer defects/oxygen vacancies, resulting in less structural disorder and/or DWF than in the SSO host matrix.^35^ This phenomenon follows from the fact that the NN Sr–O shell around Sr sites in the SSO: xEu^3+^ phosphors has fewer defects/oxygen vacancies, so the DWF is smaller than that of SSO (Table 3). As stated above, the defects or oxygen vacancies that act as trap centers are primarily attributed to the presence of the luminescent activators^13–15^, but as shown in the lower inset in Fig. 3b, the maximum intensity of the feature associated with the NN Sr–O bond length in the FT spectra of the SSO:xEu^3+^ phosphors is slightly greater than that of SSO. Apparently, the Sr *K*-edge EXAFS studies do not support the claim that defects or oxygen vacancies are formed by doping with luminescent Eu activators, which critically determine the PL property in SSO:xEu^3+^ phosphors^13–15^.

**Table 3:** Parameters obtained by best-fitting of the Sr *K*-edge EXAFS data in *R*-space mode from ~ 1.5 to 3.0 Å: nearest-neighbor (NN) coordination number (N_1_/N_2_), Sr_1_–O/Sr_2_–O bond length (R_1_/R_2_), and mean square fluctuation of the Debye–Waller factor (DWF, σ_1_^2^/σ_2_^2^) at Sr_1_/Sr_2_ sites in the SSO:xEu^3+^ phosphors and SSO.

Sample	${\text{N}}_{{1}} /{\text{N}}_{{2}}$	${\text{R}}_{{1}} /{\text{R}}_{{2}}$(Å)	${\upsigma }_{{1}}^{{ 2}} /{\upsigma }_{{2}}^{{ 2}}$(× 10^–2^ Å^2^)
1.0%	${10}/{9}$	${2}{{.92 \pm 0}}{.03}/{2}{{.68 \pm 0}}{.03}$	${2}{{.5 \pm 0}}{.1}/{2}{{.6 \pm 0}}{.1}$
2.0%	${10}/{9}$	${2}{{.92 \pm 0}}{.03}/{2}{{.69 \pm 0}}{.03}$	${2}{{.6 \pm 0}}{.1}/{2}{{.7 \pm 0}}{.1}$
5.0%	${10}/{9}$	${2}{{.90 \pm 0}}{.03}/{2}{{.66 \pm 0}}{.03}$	${2}{{.5 \pm 0}}{.1}/{2}{{.7 \pm 0}}{.1}$
SSO	${10}/{9}$	${2}{{.89 \pm 0}}{.03}/{2}{{.66 \pm 0}}{.03}$	${2}{{.6 \pm 0}}{.1}/{2}{{.8 \pm 0}}{.1}$

**Figure 4:**
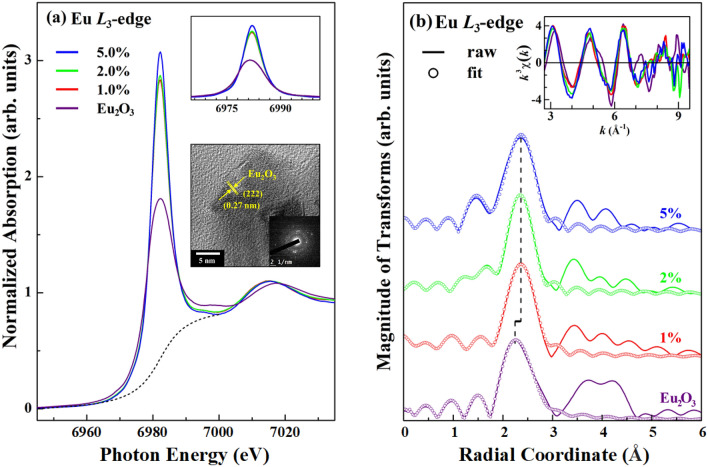
(**a**) Normalized Eu *L*_3_-edge XANES spectra of SSO:xEu^3+^ phosphors (x = 1.0, 2.0 and 5.0%) and reference Eu_2_O_3_. The upper inset shows a magnification of the near-edge features from ~ 6,960 to 7,000 eV after subtracting the arctan background from the main near-edge feature. The lower inset shows the HRTEM image of the cubic Eu_2_O_3_ phase with the presence of *d*-spacing (222) in the SSO:xEu^3+^ (x = 5.0%) matrix, and its inset shows the corresponding selective area electron diffraction pattern. (**b**) FT spectra of *k*^3^χ data of SSO:xEu^3+^ phosphors at the Eu *L*_3_-edge from *k* = 2.7 to 9.5 Å^−1^. The solid profile was obtained from raw data, whereas the circular marks represent the best fit for the first coordination shell. The inset presents Eu *L*_3_-edge EXAFS *k*^3^χ data of the SSO:xEu^3+^ phosphors and Eu_2_O_3_.

Figure 4a displays XANES spectra at the Eu *L*_3_-edge of the SSO:xEu^3+^ phosphors (x = 1.0, 2.0 and 5.0%) and Eu_2_O_3_, obtained in total fluorescence yield mode. The upper inset shows the magnification of the near-edge feature from ~ 6,960 to 7,000 eV following subtraction of the arctan background from the near-edge feature, as indicated by the dashed line in Fig. 4a. Clearly, the XANES spectra of SSO:xEu^3+^ phosphors yield a sharp line-shaped feature at the Eu *L*_3_-edge, which is almost identical to but much stronger than that of pure Eu_2_O_3_, demonstrating the formation of Eu_2_O_3_-like structures rather than Eu substitution at Sr sites in the SSO:xEu^3+^ phosphors reported elsewhere^16–19,36,37^. For confirmation of the interstitial Eu_2_O_3_-like structures in the SSO:xEu^3+^ matrix, we conducted HRTEM measurements and observed the cubic Eu_2_O_3_ phase with the presence of *d*-spacing (222) in the SSO:xEu^3+^ (x = 5.0%) matrix, as presented in the lower inset of Fig. 4a, further supports the result of the Eu *L*_3_-edge XANES spectra of the SSO:xEu^3+^ phosphors. Nevertheless, previous studies have established according to the dipole-transition selection law that the two resolvable main features of Eu *L*_3_-edge XANES at ~ 6,975 and 6,983 eV are attributable to the 2*p*_3/2_ → 5*d* electron transitions of Eu^2+^ and Eu^3+^ ions, respectively, in aqueous solution^38^ and in milled SrAl_2_O_4_:Eu^39^. Evidently, as shown in Fig. 4a, the single sharp and strong feature at ~ 6,982 eV corresponds to the transition of electrons from the 2*p*_3/2_ to 5*d* unoccupied states of Eu^3+^ ions in the SSO:xEu^3+^ phosphors, and this feature is much more intense than that in Eu_2_O_3_, particularly at the highest Eu concentration (x = 5.0%) in the SSO:xEu^3+^ phosphors. The strong intensity of the near-edge feature (many more unoccupied Eu 5*d* states) is caused by the transfer of electrons from the Eu 5*d* orbitals in the Eu_2_O_3_-like structures (or Eu^3+^–O^2−^ associations) to ^5^D_0_ levels in the SSO:xEu^3+^ phosphors, consistent with the model presented in Fig. 2b. The integrated intensity of the Eu *L*_3_ near-edge feature of the SSO:xEu^3+^ phosphors was obtained from the area in the range of 6,960 to 7,000 eV, as shown in the upper inset of Fig. 4a. Importantly, the integrated intensity of the Eu *L*_3_ near-edge feature (19.32 → 19.52 → 20.19) varies in proportion to the Eu-doping concentration in a manner similar to the variation with the PL intensity ratio, I_rat_ = I_EDT_/I_MDT_ (1.29 → 1.57 → 1.61), as discussed above. A higher Eu^3+^ activator content yields more Eu_2_O_3_-like structures in the SSO:xEu^3+^ phosphors and thereby more hypersensitive EDT, thus facilitating the transfer of electrons from Eu^3+^ 5*d* states to the ^5^D_0_ level and yielding higher PL emissions in the SSO: xEu^3+^ phosphors, as obtained/proposed in Fig. 2a,b.

To conclusively elucidate the formation by Eu^3+^ ions of interstitial Eu_2_O_3_-like structures, rather than the substitution of these ions at Sr sites in the SSO:xEu^3+^ phosphors, Fig. 4b shows the FT spectra of *k*^3^χ data of the SSO:xEu^3+^ phosphors (x = 1.0, 2.0 and 5.0%) and Eu_2_O_3_ from *k* = 2.7 to 9.5 Å^−1^ at the Eu *L*_3_-edge. The inset presents the Eu *L*_3_-edge EXAFS *k*^3^χ data for the SSO:xEu^3+^ phosphors and Eu_2_O_3_. The feature in the FT spectra at ~ 2.4 Å corresponds to the NN Eu–O bond length, and the second main feature at ~ 3.2–3.3 Å corresponds to the Eu–Eu bond length. The first (second) main FT feature of SSO:xEu^3+^ reflects a slightly longer (shorter) Eu–O (Eu–Eu) bond length in the SSO:xEu^3+^ phosphors than in Eu_2_O_3_. The general FT spectra at the Eu *L*_3_-edge of SSO:xEu^3+^ phosphors and Eu_2_O_3_ exhibit similar line shapes of FT features in Fig. 4b, confirming that the local atomic structures of Eu^3+^ ions in the SSO:xEu^3+^ phosphors are comparable to that of the cubic phase of Eu_2_O_3_. To quantitatively elucidate the local atomic structures around Eu^3+^ ions in SSO:xEu^3+^ phosphors and Eu_2_O_3_, the fitting results concerning the NN coordination number (N), Eu–O bond length (R) and corresponding DWF (σ^2^) were also obtained using the Athena program package^33,34^ and are presented in Table 4. Although the amount of Eu_2_O_3_-like structures in the host matrix is very small and not detectable by long-range sensitive XRD measurements, as presented in Fig. 1c, they were indeed incorporated as interstitial Eu_2_O_3_-like structures in the host matrix, as revealed by the short-range sensitive XANES, EXAFS and HRTEM techniques, as shown in Fig. 4a,b, respectively. Notably, the general XANES line-shapes/FT spectral features at the Eu *L*_3_-edge in Fig. 4a,b and the results concerning the local atomic structures (N, R and σ^2^) in Table 4 clearly differ from the Sr *K*-edge XANES line-shapes/FT spectral features in Fig. 3a,b and Table 3, conclusively revealing that the Eu^3+^ ions formed Eu_2_O_3_-like structures in the host matrix. No evidence supports substitution of the Eu^3+^ ions for the Sr^2+^ ions at the Sr_1_ and/or Sr_2_ sites in the SSO:xEu^3+^ phosphors^16–19,36,37^. Ultimately, the covalent bond energy of Eu–O (2.95 eV) exceeds that of Sr–O (2.66 eV),^18^ implying that Eu forms much stronger bonds with neighboring O atoms than does Sr, so Eu_2_O_3_-like interstitial structures are formed preferentially in the SSO:xEu^3+^ matrix.

**Table 4:** Parameters obtained by best-fitting the Eu *L*_3_-edge EXAFS data in *R*-space mode from ~ 1.7 to 3.0 Å: NN coordination number (N), Eu–O bond length (R) and corresponding DWF (σ^2^) at the Eu ions in the SSO:xEu^3+^ phosphors and Eu_2_O_3_.

Sample	N	R (Å)	${\upsigma }^{{2}}$(× 10^–2^ Å^2^)
1.0%	6.0 ± 0.1	2.39 ± 0.01	1.0 ± 0.1
2.0%	6.0 ± 0.1	2.38 ± 0.01	0.8 ± 0.1
5.0%	6.0 ± 0.1	2.39 ± 0.01	1.0 ± 0.1
${\text{Eu}}_{{2}} {\text{O}}_{{3}}$	6.0	2.33 ± 0.01	1.3 ± 0.1

**Figure 5:**
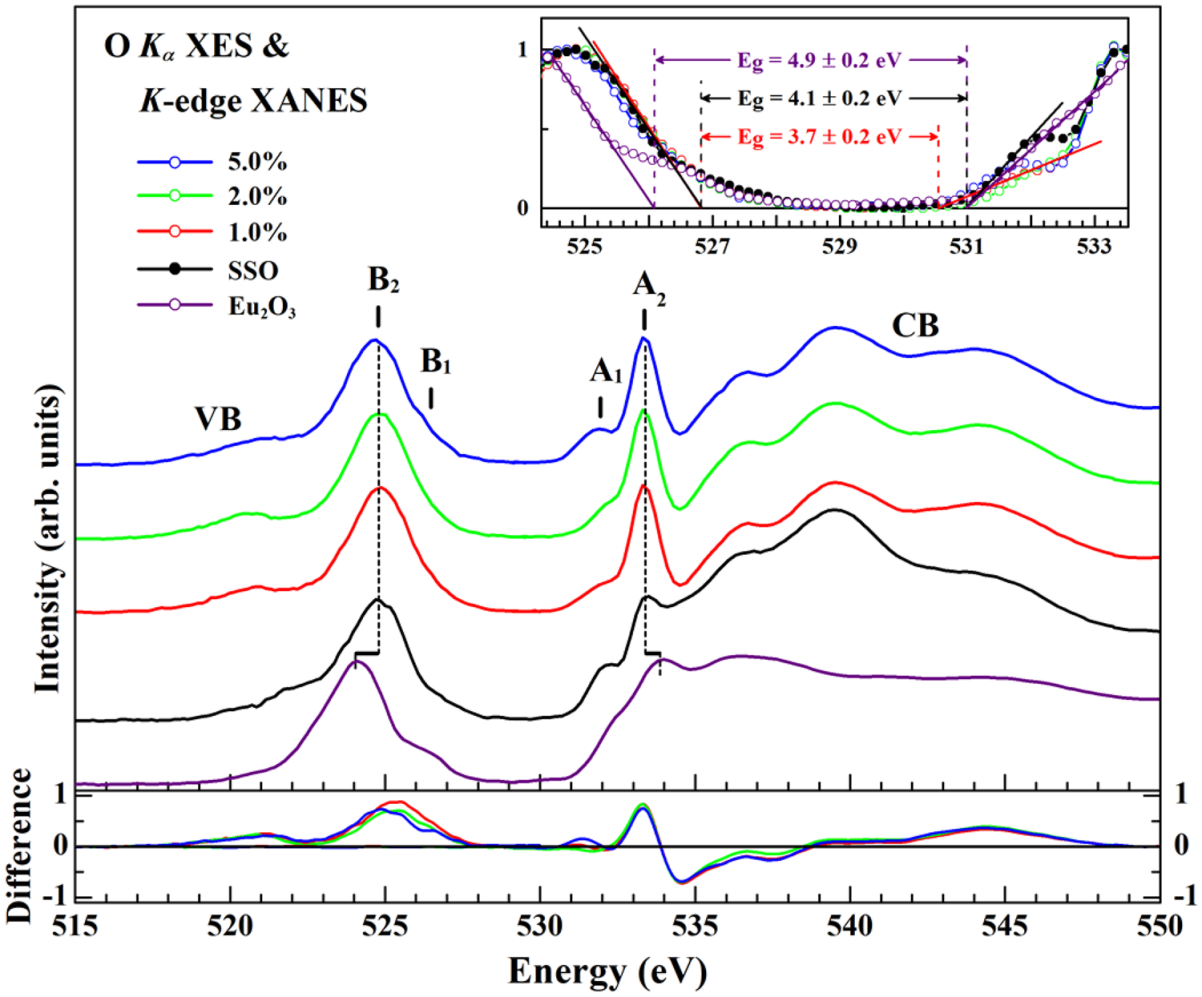
Normalized O *K*-edge XANES and *K*_α_ XES spectra of SSO:xEu^3+^ phosphors (x = 1.0, 2.0 and 5.0%), SSO and Eu_2_O_3_. The inset shows the CBM and VBM, as indicated by the solid lines and obtained by extrapolating the leading edges in the XANES and XES spectra to the baseline (guided by eye). The lower panel shows the different spectra of O *K*-edge XANES and *K*_α_ XES between SSO:xEu^3+^ phosphors and SSO.

As discussed above, when the phosphor is excited by a suitable wavelength/energy, it can exhibit high PL intensity with desirable emission chromaticity, which is strongly related to the electronic structures between the luminescent activators and the host matrix. To reveal the role of the Eu^3+^-activator in SSO:xEu^3+^ phosphors, Fig. 5 shows the normalized O *K*-edge XANES and *K*_α_ XES spectra there of (x = 1.0, 2.0 and 5.0%) and references SSO and Eu_2_O_3_. These spectra show the correlations among PL, DOSs near the VBM/CBM and the bandgap of the SSO:xEu^3+^ phosphors, as well as the differences between the electronic structures and band gap of the SSO:xEu^3+^ phosphors from those of the SSO host lattice. As displayed in Fig. 5, based on the first-principles calculations by Pan et al.^19^, the DOSs near/at the CBM (features A_1_ and A_2_) of SSO mostly involve O 2*p* and Si 3*p* states, while the DOSs near/at the VBM (features B_1_ and B_2_) are dominated by O 2*p* and Si 3*p* states; the Sr 4*p* states at ~ 15 eV are far below the VBM of SSO. The α′ and β phases of SSO generally exhibit similar total and partial DOSs in the lattice. Theoretical calculations based on first principles indicate that Eu_2_O_3_ can exist as three stable structures (cubic, monoclinic and hexagonal phases) under ambient pressure. The novel correlation property of Eu_2_O_3_ is strongly related to *f*-*f* interactions and highly localized electrons in the 4*f* states of Eu ions in the compound, generating several metastable electronic configurations that depend on the partial occupation of 4*f* states.^40^ Systematic studies of electronic band structures using the *GW* with the Hubbard *U* corrected local-density approximation have shown that the electronic structures of Eu_2_O_3_ in its three phases are similar, but the positions of occupied/unoccupied Eu 4*f* and 5*d* states near the VBM/CBM of Eu_2_O_3_ vary among the phases because the Eu 4*f*/5*d* states have different orbital symmetries.^40^ Nonetheless, as shown in Fig. 5, the O *K*-edge XANES absorption features (between features A_1_ and A_2_) of Eu_2_O_3_ at ~ 531.8 and 533.5 eV are attributed to unoccupied O 2*p*–Eu 4*f* and O 2*p*–Eu 5*d* hybridized states, respectively, above/at the CBM. The Eu 4*f* and 5*d* states strongly overlap with each other, and the Eu 4*f* state is below the energy level of the 5*d* states. The O *K*_α_ XES features (between features B_1_ and B_2_) at ~ 526.5 and 524.8 eV are attributed to occupied O 2*p*–Eu 4*f* hybridized states, and the B_1_ feature is predominately occupied by Eu 4*f* states below/at the VBM of Eu_2_O_3_^40,41^. The A_2_ and B_2_ features of Eu_2_O_3_ appear to be shifted to higher energy than those of SSO:xEu^3+^ phosphors and SSO, as clearly indicated by the dashed line shown in Fig. 5. The general line shapes of the emission and absorption features, especially of the A_1_ and A_2_ absorption features in the SSO:xEu^3+^ phosphors, differ from those of the SSO host due to the incorporation of Eu_2_O_3_-like structures in the host matrix. Clearly, the two main absorption features, A_1_ and A_2_, of the SSO:xEu^3+^ phosphors herein are associated with the O 2*p*, Si 3*p* and O 2*p*–Eu 4*f*/5*d* hybridized states, whereas the B_1_ and B_2_ emission features are primarily associated with occupied O 2*p*, Si 3*p* and O 2*p*–Eu 4*f* hybridized states. The inset in Fig. 5 represents a well-defined bandgap of E_g_ by the solid lines that are obtained by extrapolating the leading edges in the O *K*-edge XANES and O *K*_α_ XES spectra to the baselines,^42,43^ corresponding to the positions of the CBM and the VBM of SSO:xEu^3+^ phosphors, SSO and Eu_2_O_3_, respectively. In the inset, the maximum intensity of the A_2_/B_2_ feature of XANES/XES is normalized to unity. The energy separation between the CBM and the VBM yields E_g_ values of ~ 3.7 ± 0.2, 4.1 ± 0.2 and 4.9 ± 0.2 eV for SSO:xEu^3+^ phosphors, SSO and Eu_2_O_3_, respectively. The values of E_g_ for the SSO (~ 4.1 eV) and Eu_2_O_3_ (~ 4.9 eV) references are reasonably comparable with the following values in the literature mentioned above: ~ 4.49/4.11 eV for corresponding α′-phase/β-phase SSO^19^, ~ 4.12 eV for the mixed α′- and β-phases of SSO^32^ and 4.4 eV for Eu_2_O_3_^44^. However, the combination of O *K*-edge XANES and *K*_α_ XES spectra demonstrated that the value of E_g_ for the SSO:xEu^3+^ phosphors remained almost constant with Eu doping, but it was smaller (~ 3.7 eV) than that of SSO (~ 4.1 eV), indicating that the O 2*p–*Eu 4*f*/5*d* hybridized states of Eu_2_O_3_-like structures in the SSO:xEu^3+^ phosphors contribute considerably to the DOSs within the bandgap (or near/at the CBM and the VBM, respectively) of SSO. Notably, the threshold feature A_1_ in the O *K*-edge XANES shifted slightly toward lower energies, while the threshold feature B_1_ in the O *K*_α_ XES spectra remained fairly unchanged upon doping with Eu; accordingly, the unoccupied O 2*p–*Eu 4*f*/5*d* (occupied O 2*p–*Eu 4*f*) hybridized states are significantly distributed near/at the CBM (VBM), decreasing the bandgap of SSO:xEu^3+^ phosphors of ~ 3.7 eV, which is lower than that of the SSO host matrix (~ 4.1 eV), as clearly observed in the magnified scale in the inset of Fig. 5.

To reveal the enhancement of DOSs within the band gap of SSO:xEu^3+^ phosphors that is caused by doping of the SSO host matrix with Eu, the lower panel in Fig. 5 displays the difference between unoccupied/occupied states of the CB/VB of SSO:xEu^3+^ phosphors and those of the SSO. As revealed by the XRD data in Fig. 1c, the minor/impurity SCO phase may also contribute to the O *K*-edge XANES and *K*_α_ XES spectra of the SSO:xEu^3+^ phosphors and the SSO, but its intensity can be treated as an equal quantity, canceling out in the different XANES and XES spectra, as shown in the lower panel. A large difference in the O *K*-edge XANES and *K*_α_ XES spectra around the A_1_ (B_1_) and A_2_ (B_2_) features was clearly observed between the spectra near/at the CBM (VBM) of the SSO:xEu^3+^ phosphors and the SSO. The enhanced features near/at the CBM (VBM) in the right (left) lower panel arise from the increased density of the unoccupied O 2*p*–Eu 4*f*/5*d* states (occupied O 2*p*–Eu 4*f* states). In particular, the large enhancement of the A_2_ feature (O 2*p*–Eu 5*d* states) reveals an increase in the DOSs in the bandgap of SSO:xEu^3+^ phosphors upon doping with Eu. The electron excitation from the VB to the CB by excitation by UV light (λ = 250 nm, ~ 4.96 eV) exceeds the energy gap E_g_ (~ 3.7 eV) of SSO:xEu^3+^ phosphors, yielding extra free electrons and holes at the CB and VB, respectively, owing to incorporation of Eu_2_O_3_-like structures in the host matrix. The increase in the intensities of the features in the O *K*-edge XANES and *K*_α_ XES spectra demonstrate the contribution of O 2*p–*Eu 4*f*/5*d* hybridized states (mostly from unoccupied Eu 5*d* states at the CB and occupied O 2*p–*Eu 4*f* states at the VB, respectively) in the bandgap of the host matrix. As shown in Fig. 2b, the O 2*p–*Eu 4*f*/5*d* (major of Eu 5*d* states, Eu^3+^–O^2−^ associations) above/at CBM likely act as donor levels for the nonradiative transfer of electrons to ^5^D_0_ levels, generating unoccupied states, as consistently observed at the O *K*-edge and Eu *L*_3_-edge XANES (Figs. 4a, 5), and yielding intra 4*f*–4*f* electronic transitions followed by excited ^5^D_0_ → ^7^F_J_ (J = 1, 2, 3 and 4) transitions, which enhance the PL of SSO:xEu^3+^ phosphors upon Eu^3+^-doping. Again, based on the above results, the CT band plays a critical role in the radiative emission of Eu^3+^-activators because Eu_2_O_3_-like structures formed interstitially in the SSO:xEu^3+^ matrix rather than at polyhedral SrO_10_/SrO_9_ sites in the SSO:xEu^3+^ matrix^1,3,18^. Additionally, due to the slightly varied crystal field splitting of Eu activators in the matrix, the PL property of SSO:xEu^3+^ phosphors is caused by the Eu_2_O_3_-like structures, yielding all of the PL emission features (~ 590, 613, 652 and 703 nm) at slightly higher wavelengths than those of Eu_2_O_3_ thin films (~ 583, 611, 648 and 694 nm)^31^ and at lower wavelengths than those of Sr_1.9_SiO_4_:0.1Eu powder (~ 592, 620, 654 and 704 nm)^45^. Since Eu_2_O_3_-like structures act as interstitial sites in the SSO:xEu^3+^ phosphors, SSO is a host material with a large bandgap and stable lattice, making it a suitable host for accommodating Eu^3+^-activator phosphors.

## Conclusion

In conclusion, PL measurements show that the wavelengths of the emission spectra do not significantly vary with Eu^3+^ doping concentration in SSO:xEu^3+^ phosphors. However, the PL intensity increases with increasing Eu^3+^ doping concentration. The PL intensity is associated with the α′-SSO/β-SSO phase weight ratio in the SSO:xEu^3+^ phosphors. At higher Eu^3+^ contents, the luminescence is stronger because more Eu_2_O_3_-like structures are present in the host matrix, favoring the nonradiative transfer of electrons from Eu 5*d* states above/at the CBM to the ^5^D_0_ level, which observably increases the absorption intensity at Eu *L*_3_-edge XANES spectra of SSO:xEu^3+^ phosphors. Furthermore. The results of the O *K*-edge XANES and *K*_α_ XES spectra clearly demonstrate that the unoccupied O 2*p–*Eu 4*f*/5*d* and occupied O 2*p–*Eu 4*f* states within the bandgap (or near/at the CBM and the VBM) of the matrix promote the nonradiative transfer of electrons from the O 2*p–*Eu 4*f*/5*d* hybridized states (mostly Eu 5*d* states above/at the CBM) to the ^5^D_0_ level, facilitating electronic transitions from the excited ^5^D_0_ to ^7^F_J_ (J = 1, 2, 3 and 4) levels. This CT process is primarily responsible for the enhancement of the PL of SSO:xEu^3+^ phosphors with increased Eu^3+^-doping in the matrix. Significantly, this study demonstrates that the Eu activators (or Eu^3+^–O^2−^ associations) interact weakly with the host matrix, which provides sufficient room to accommodate the Eu^3+^ dopants as luminescence centers without substitution at the Sr sites in SSO: xEu^3+^ phosphors.

## Experimental methods and characterizations

The SSO:xEu^3+^ phosphors in the α′ phase and the β phase with various Eu^3+^ concentrations (x = 1.0, 2.0 and 5.0%) were synthesized using a sol–gel route at ~ 1,100–1,200 °C with SCO, SiO_2_ and Eu_2_O_3_ as starting materials. In this sol–gel process, stoichiometric ratios of SCO, SiO_2_ and Eu_2_O_3_ were taken in the solvent into an aqueous solution (called sol). The solutions were then hydrolyzed and condensed. The slow aggregation among colloidal particles formed a three-dimensional network structure with a poorly liquid gelatin (called gel). The resulting gel was then dried and sintered at high temperature (~ 1,100–1,200 °C) to form the final powder product of SSO:xEu^3+^. The formed crystal structures were mostly SSO with a tiny amount of SCO phases. The atomic structures of the (orthorhombic) α′ and (monoclinic) β phases of the SSO matrix in Fig. 1a, b were drawn using VESTA software^46^ after considering the SSO raw crystallographic files (https://www.materialsproject.org/). XRD patterns were obtained using Cu *K*_α_ (λ = 1.5418 Å) radiation at 40 kV. Energy-dispersive X-ray spectroscopy measurements for elemental mapping analysis showed the presence of Eu, but it is very difficult to obtain an accurate quantitative number for Eu concentrations due to the very low Eu content in each SSO:xEu^3+^ phosphor. Hence, the “Eu-content %” mentioned in the text is the stoichiometric % taken during the synthesis process. The PL spectra were recorded at room temperature within the wavelength range of ~ 200–800 nm on a Hitachi F-4500 fluorescence spectrophotometer equipped with a 150 W Xe lamp, with emission upon excitation at a wavelength of 250 nm (~ 4.96 eV). Eu *L*_3_-, Sr *K*-edge XANES/EXAFS and O *K*_α_ XES spectra were measured at the Taiwan Photon Source (TPS) 44A and 45A-undular beamlines^47^; O *K*-edge XANES was performed at the Taiwan Light Source (TLS) 20A beamline of the National Synchrotron Radiation Research Center, Taiwan. The O *K*_α_ XES spectra were obtained at an excitation energy of 550 eV with a resolution of better than 0.2 eV; the intensity of the XES features in the energy range between ~ 517 and 515 eV was normalized to unity. The O *K*-edge XANES spectra were obtained in fluorescence yield mode with a resolution greater than 0.1 eV and were normalized to the incident beam intensity following pre-edge background subtraction, with the area under the spectra in the energy range between ~ 550 and 555 eV fixed. The energy resolutions of Eu *L*_3_- and Sr *K*-edge XANES were set to ~ 0.5 and 1 eV, respectively.
